# The impact of the COVID-19 pandemic on eating disorders risk and symptoms: a retrospective study

**DOI:** 10.1186/s13052-023-01443-6

**Published:** 2023-04-26

**Authors:** Elisabetta Straface, Isabella Tarissi De Jacobis, Teresa Capriati, Italo Pretelli, Annalisa Grandin, Cristina Mascolo, Rosa Vona, Lucrezia Gambardella, Camilla Cittadini, Alberto Villani, Maria Rosaria Marchili

**Affiliations:** 1 Center for Gender-Specific Medicine, Biomarkers Unit, Istituto Superiore di Sanità, Viale Regina Elena 299, Rome, 00161 Italy; 2 Emergency Acceptance and General Pediatric Department, Bambino Gesù Children’s Hospital, IRCCS, Piazza di Sant’Onofrio 4, Rome, 00165 Italy; 3 Gastroenterology and Nutritional Rehabilitation, Bambino Gesù Children’s Hospital, IRCCS, Piazza di Sant’Onofrio 4, Rome, 00165 Italy; 4 Anorexia and Eating Disorder Unit, Child and Adolescent Psichiatry Unit, Bambino Gesù Children’s Hospital, IRCCS, Piazza di Sant’Onofrio 4, Rome, 00165 Italy; 5 Pediatric Academic Department, University of Rome Tor Vergata, Via Cracovia 50, Rome, 00133 Italy; 6 Biomarkers Unit, Center for Gender-Specific Medicine, Istituto Superiore di Sanità, Viale Regina Elena, Rome, 299 - 00161 Italy

**Keywords:** Covid-19, Pandemic, Eating disorders, Comorbidity

## Abstract

**Background:**

Social distancing and quarantine imposed by the authority during the COVID-19 pandemic caused restrictions, which had a negative impact on eating behavior, especially among adolescents. We proposed a retrospective study aimed to evaluate the effect of the COVID-19 pandemic on eating disorders risk and symptoms.

**Methods:**

In this study, a group of 127 pediatric patients (117 females and 10 males) with eating disorders admitted to the Bambino Gesù Children’s Hospital of Rome (Italy), in the period between August 2019 and April 2021, was analyzed. All patient data were collected from patients’ electronic medical records.

**Results:**

We found that 80.3% of patients were at the onset of eating disorders and that 26% of patients had familiarity for psychotic disorders. Often these patients had comorbidities and alterations in blood parameters such as leukocytopenia, neutropenia, hypovitaminosis and hormonal problems that could affect their future.

**Conclusions:**

Our findings could provide a framework for developing clinical and educational interventions to mitigate the short- and long-term negative impact of the pandemic on adolescent future health.

**Supplementary Information:**

The online version contains supplementary material available at 10.1186/s13052-023-01443-6.

## Background

The lockdown imposed by the authority during COVID-19 pandemic, although effective in reducing the transmission of the infection, resulted in marked changes in the lifestyle of the general population (e.g., social distancing, isolation and quarantine, closure of the schools, businesses, gyms, and restaurants). Early reports suggest that social distancing and quarantine are having adverse consequences on mental health including high levels of stress, anxiety, depression, sleep disturbances, and in particular eating disorders (EDs) [1]. It has been observed that individuals with pre-existing EDs and / or obesity may be particularly vulnerable, due to the associated psychiatric comorbidities and metabolic anomalies [2]. EDs are serious mental health disorders that cause impairments in physical health, development, cognition, and psychosocial function and can go undetected for months or years. They are common in childhood and adolescence and often are followed by comorbid disorders such as anxiety, self-harm, and substance use [3, 4]. Frequently, they have been associated with both suicidal and para-suicidal behaviors, as well as suicide [5].

The presence of EDs is mainly attributed to (i) family environment (e.g., parental psychiatric disorders, prenatal maternal stress); (ii) restriction to daily activities and movements; (iii) excessive exposure to harmful eating patterns on social media, emotional distress, fear of contagion, and low access to care [3]. According to the Diagnostic and Statistical Manual of Mental Disorders, 5th Edition (DSM-5) [6], EDs commonly observed in children and adolescents can be classified in anorexia nervosa (AN), bulimia nervosa (BN), binge-eating disorder (BED) and avoidant/restrictive food intake disorder (ARFID). The first is an eating disorder characterized by pathological problems of weight and shape that lead to reduced food intake and consequent low weight [7]. The second is an ED characterized by recurrent episodes of binges eating followed by compensatory behaviors (e.g., self-induced vomiting, misuse of laxatives, diuretics, or other drugs, fasting, excessive exercise) [8]. The third is characterized by recurrent episodes of bingeing associated with distress overeating or eating in the absence of hunger [9]. The last is characterized by avoidance or restriction of food, motivated by fear of the adverse consequences of eating or a lack of interest in eating [10].

Some studies state that, with respect to men, women have a higher risk of developing health problems related to EDs. The ratio of women to men for EDs has been reported to range from 4: 1 to 10: 1 and significantly increases during puberty [6]. A growing number of studies conducted during the pandemic have documented a worsening of eating disorder symptoms and syndromes in a variety of population groups [11–15].

Some studies showed increased eating restriction in people with AN, and more frequent binge-eating episodes in those with BN or BED [16–18]. In the first wave of the pandemic, an increase in symptoms such as anxiety, depression and eating disorders has been observed mainly in adolescents with pre-existing EDs. Instead, during the second and subsequent waves of the pandemic a surge in suicidal ideation and suicide attempts among adolescents has been reported in many countries [19].

Given the severity of these disorders, our study is aimed to explore, in a sample of young Italian peoples, the effect of the COVID-19 pandemic on eating disorders risk and symptoms.

This is a retrospective study based on data from the medical records of adolescents admitted to the Bambino Gesù Children’s Hospital of Rome (Italy) in the period between August 2019 and April 2021. Our findings could provide a framework for developing clinical and educational interventions to mitigate the short- and long-term negative impact of the pandemic on the future health of adolescents.

## Methods

**Fig. 1:**
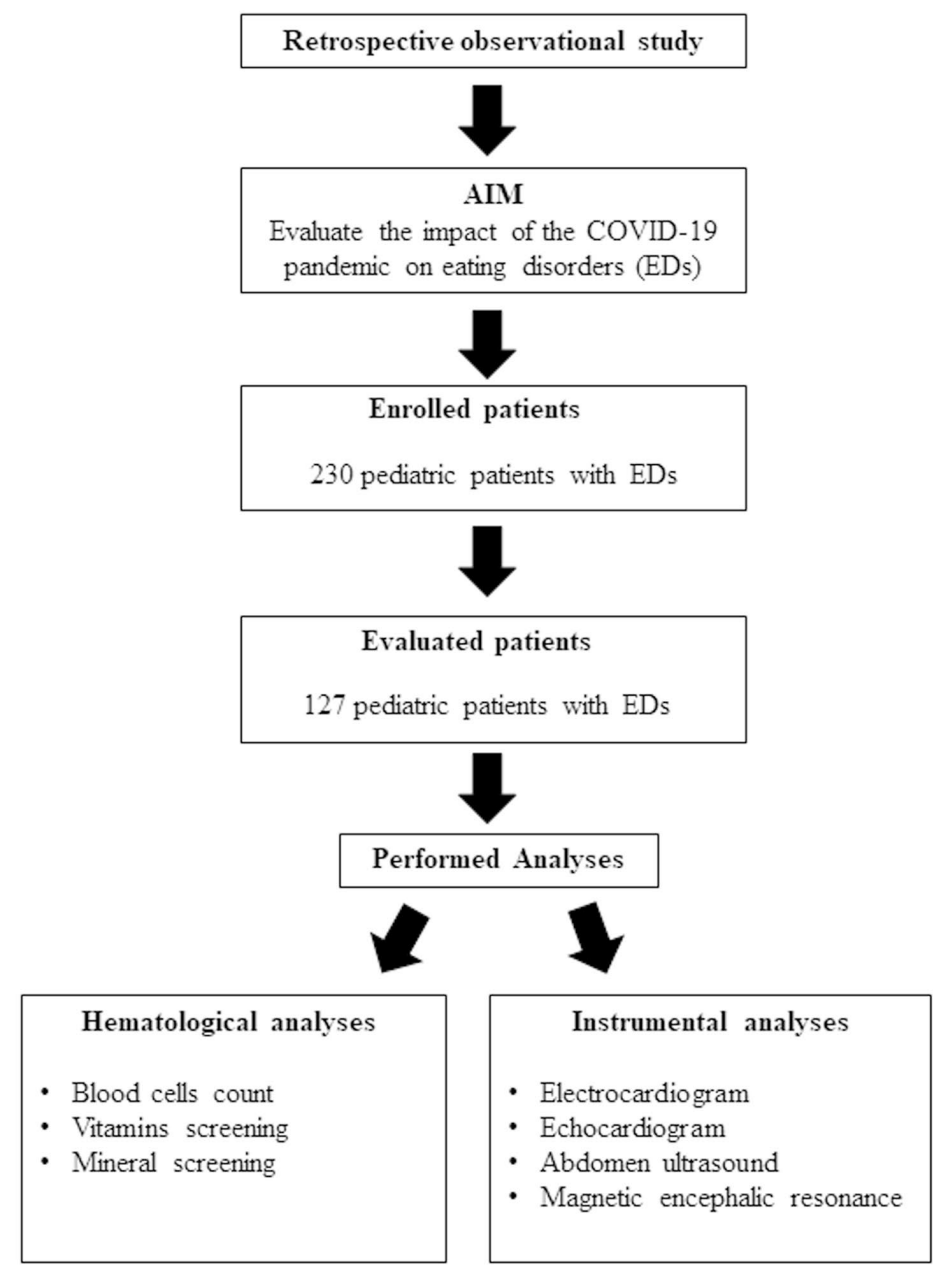
A workflow chart of the study

### Patients

This is a retrospective observational study of a group of pediatric patients with EDs admitted to the Bambino Gesù Children’s Hospital of Rome (Italy) in the period between August 2019 and April 2021. In this study 230 patients with EDs were enrolled, but only 127 patients (117 females and 10 males) who had a follow-up after 1 month, 3 months and, in some cases, 12 months of antipsychotic therapy, were evaluated. Medical history, comorbidities, and laboratory data were obtained from patients’ electronic medical records related to admission (T0) and follow-up.

The study was approved by ethics committee of the Bambino Gesù Children’s Hospital of Rome (approval number: 2526-OPBG-2021). A written informed consent was obtained from adult patients and parents of patients under the age of 18.

### Procedures

During hospitalization, patients were subjected to (i) laboratory analyses for the evaluation of blood cells count and screening for specific vitamins and mineral deficiencies; and (ii) electrocardiogram, echocardiogram, abdomen ultrasound, and magnetic encephalic resonance.

All methods were performed in accordance with the ethical standards as laid down in the Declaration of Helsinki and its later amendments or comparable ethical standards.

### Treatments

After diagnosis, the patients were treated with antipsychotics drugs as follows: 23 patients with antipsychotics; 80 with antipsychotics in combination with serotonin uptake inhibitors; 9 with antipsychotics in combination with benzodiazepines; 2 with antipsychotics in combination with anxiolytics; 1 with antipsychotics in combination with lithium; and 3 with serotonin uptake inhibitors. Moreover, 9 patients did not receive any drug treatment.

### Statistical analysis

Correlations were evaluated by using Pearson correlation (r correlation coefficient). To test the probability of significant differences, individual group comparisons were evaluated using Bonferroni’s test. p < 0.05 values were considered statistically significant. A two-way analysis of variance using JMP 10 software (SAS Institute srl, Milan, Italy) was used to connect changes observed in ED patients with some comorbidities.

## Results

### General characteristics of partecipants

A total of 127 patients (117 females and 10 males) with EDs, aged between 10 and 18 years, were included in this study. Most of the patients (74%) lived on central Italy while a small percentage (26%) in southern Italy. Patients were hospitalized for an average of 24 days (range 3–83). A very low percentage of patients included in this study was anorexic (5.5%) or had avoidant restrictive food intake disorder (ARFID, 4.7%), while 89.8% of patients had unspecified eating disorders. Except two patients who had a weight of 76.2 and 81.5 kg respectively, and a body mass index (BMI) of 24.3 and 28.2 Kg/m^2^ respectively, the other patients had a median weight value of 37.2 Kg (range 22.5–52.6 Kg) and median BMI value of 14.96 Kg/m^2^ (range 11.3–28.2 Kg/m^2^). As shown in the Tables 1 and 102 patients (94 females and 8 males) were at the first onset of the disease, while 25 patients (23 females and 2 males) had clinical relapse. In addition, 79 patients (75 females and 4 males) had previously been hospitalized and 33 patients (32 females and 1 male) had familiarity for psychotic disorders. Specially, they were familiar for depression (7%), anxiety (1.6%), EDs (4.7%), and psychosis (4%). Moreover, the patients had familiarity for diabetes (3.9%); hyperthyroidism (3.1%) and hypothyroidism (2.4%). During hospitalization, echocardiographic examination highlighted pericardial effusion in 10 patients (7.8%) and pericardial cleavage in 7 patients (5.5%). Abdominal echography in 9 patients revealed pathologies such as angioma, calculosis of the cholecode and modest fluid flap in the pelvic cavity. Nuclear magnetic resonance of the brain resulted in a pathological outcome in only 7 patients.

**Table 1:** General characteristics of ED patients

Characteristics	Patients (n = 127)
Age, median (range)- years	14 (range 10–18)
Sex	117 (92%) Females
	10 (7.8%) Males
Hospitalization, median (range)-days	24.13 (3–83)
Body weight, median (range)-Kg	37.2 (range 22.5–52.6)
BMI, median (range)- Kg/m^2^	14.96 (range 11.3–28.2)
Patients first onset of EDs	102 (80.3%)
Patients with clinical relapse	25 (19.7%)
Patients previously hospitalized	79 (62.2%)
Familiarity for psychotic disorders	33 (26%)
Familiarity for diabetes	5 (3.9%)
Familiarity for hyperthyroidism	4 (3.1%)
Familiarity for hypothyroidism	3 (2.4%)

BMI, body mass index; EDs, eating disorders

### Comorbidity in ED patients

As listed in the Table 2, patients analyzed in this study had comorbidity.

A high percentage of females (64%) had amenorrhea. All patients had low lymphocytopenia, while 38% of patients had neutropenia and 13.4% of patients had thrombocytopenia. Many patients had bradycardia (60.6%) and hypovitaminosis (93.5%), while a small percentage (7%) had hypercreatinemia and hyperazotemia. In addition, these patients also had disorders such as depression (14%), anxiety (3.9%), psycosis (4.7%), specific learning disorder (3.9%) or multiple psychiatric disorders (13.4%). Two patients had also attempted suicide.

**Table 2:** Comorbidities detected in ED patients

Comorbidities	Percentage of ED patients
Amenorrhea	64% of female patients
Lymphocytopenia	100%
Neutropenia	38%
Thrombocytopenia	13.4%
Bradycardia	60.6%
Hypovitaminosis	93.5%
Effusion	10.2%
Hypercreatinemia	7%
Hyperazotemia	7%
Depression	14%
Anxiety	3.9%
Psychosis	4.7%
Specific learning disorder (SLD)	3.9%
Multiple psychiatric disorders	13.4%

### Screening for vitamins and thyroid hormones

As previously reported, a high percentage of these patients had hypovitaminosis. In particular, a deficiency of vitamin A (6% of patients); vitamin B1 (7% of patients); vitamin B6 (9.4% of patients); vitamin B12 (6.3% of patients) and vitamin C (27% of patients) was detected (Table 3). Moreover, we found that vitamin D3 was deficient in the 22.8% of patients and insufficient in the 42% of patients. In addition, 56.6% of patients had high levels of ferritin, while 7.9% of patients had high levels of thyrotrophic hormone (TSH). Instead, 21% of patients had low levels of vitamin B9 (folic acid), while 21.3% of patients had low levels of thyroxine (FT4) (Table 3).

**Table 3:** Percentage of patients with variables having a different value from reference values

	Patient percentage	Median values	Reference values
Vitamin A	6%	0.5 (range 0.4–0.7)	0.7–2.8 µM/mL
Vitamin B1	7%	27.3 (range 21.3–30.3)	32–95 ng/ml
Vitamin B6	9.4%	4.6 (range 0.5–8)	8.7–27.2 ng/mL
Vitamin B12	6.3%	199.5 (range 25–287)	300–900 ng/mL
Vitamin C	27%	15 (range 3.5–24)	26.1–84.6 µM/L
Vitamin D3	22.8%	15.96 (range 4.8–19.8)	< 20 ng/ml deficient
	42%	25.3 (range 20.3–29.5)	< 30 ng/ml insufficient
Ferritin	56.6%	293 (range 155–990)	13–150 ng/mL
TSH	7.9%	5 (range 4.37–6.7)	0.51–4.3µlU/mL
Vitamin B9 (folic acid)	21%	3.54 (range 1.83–4.91)	5 -27.2 ng/mL
FT4	21.3%	0.88 (range 0.71–0.96)	0.98–1.64 ng/dL

TSH, thyrotrophic hormone; FT4, thyroxine

### Correlations between variables

Considering that all patients have lymphocytopenia as comorbidity, we correlated leukocyte number with some variables. As shown in Table 4 we found that lymphocytes number correlated significantly (p < 0.0001) with body weight (rho = 0.08); BMI (rho = 0.016); neutrophil count (rho = 0.065), vitamin B1 (rho = 0.06); vitamin B6 (0.11); vitamin D3 (rho = 0.02); ferritin (rho = 0.089); sideremia (rho = 0.012); and cholesterol (rho = 0.18). Conversely, no correlation was found between lymphocytes number and vitamin A (rho = -0.14); vitamin B12 (rho = -0.07); vitamin B9 (rho = -0.11); TSH (rho = -0.06); FT4 (rho = -0.104); and triglycerides (rho = -0.19). Moreover, to connect hematological changes detected in ED patients with comorbidity such as bradycardia, amenorrhea, anxiety, and depression, a regression analysis was performed, but no correlations were found.

**Table 4:** Correlation between variables

	Lymphocytes (rho values)	p values (<)	
Body weight	0.08	0.0001	
BMI	0.016	0.0001	
Neutrophils	0.065	0.0001	
Vitamin A	-0.14	0.0001	
Vitamin B1	0.06	0.0001	
Vitamin B6	0.11	0.0001	
Vitamin B9	− 0.11	0.0001	
Vitamin B12	− 0.07	0.0001	
Vitamin C	0.12	0.0001	
Vitamin D	0.02	0.0001	
Ferritin	0.089	0.0001	
Sideremia	0.012	0.0001	
TSH	− 0.06	0.0001	
FT4	− 0.104	0.0001	
Cholesterol	0.18	0.0001	
Triglycerides	− 0.19	0.0001	

BMI, body mass index; TSH, thyrotrophic hormone; FT4, thyroxine

### Efficacy of therapy

Antipsychotic treatment positively influenced both body weight and BMI of patients. As shown in Fig. 1, compared to T0, weight increased by 10.4% after 1 month and by 26.2% after 3 months of antipsychotic treatment. Body weight increase after 3 months was significant (p < 0.05). Similarly, the BMI increased by 12.3% after 1 month and by 15% after 3 months of treatment.

**Fig. 2:**
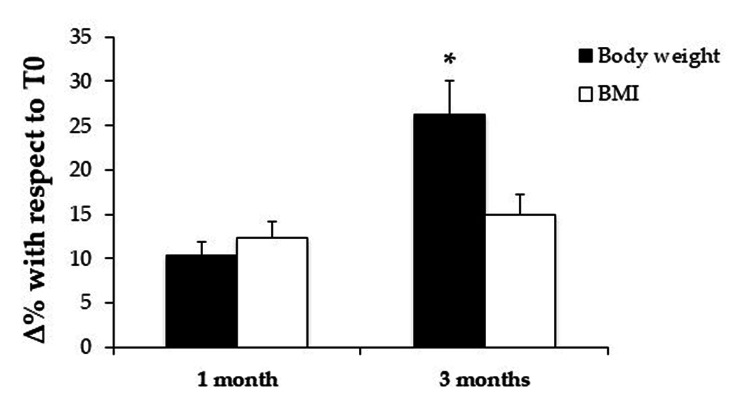
Effects of antipsychotic treatment on body weight and BMI of EDs patients. In the histogram are shown the values of body weight and BMI after 1 and 3 months of treatments with antipsychotics. The values are expressed as delta percentage compared to T0. *p < 0.05

## Discussion

The COVID-19 pandemic resulted in restrictions, which had a negative impact on physical activity and eating behavior, especially among young population. Children and adolescents affected by pre-existing eating disorders, being more sensitive to social stress [18] and having difficulties to control emotions [19], may be particularly vulnerable. In our retrospective study a group of 127 pediatrics patients with EDs admitted to the Bambino Gesù Children’s Hospital of Rome (Italy) during the COVID-19 pandemic were studied and hematologic complications (lymphocytopenia and neutropenia), amenorrhea, bradycardia, alterations in hormone status and psychiatric comorbidities (anxiety and depression) were found.

In patients with anorexia nervosa literature data associate some hematological complications with malnutrition [20] and state that leukopenia can promote an alteration of the humoral and cellular immune responses [21]. In this study we found that most of the patients analyzed suffer from hypovitaminosis due to malnutrition. Among the vitamins evaluated, vitamin C, vitamin B9 (folic acid) and vitamin D3 were deficient in a higher percentage of patients (27, 21, and 64.8% respectively). Vitamin C is an antioxidant vitamin that modulates immune cell function and supports a Th1 cytokine-mediated immune response with sufficient production of pro-inflammatory cytokines [22]. Vitamin B9 plays an essential role in the synthesis of neurotransmitters and structural elements of neurons. Its deficiency has been associated with disorders linked to mental function such as depression and cognitive function impairment [23]. Vitamin D3 plays important roles in both cell-mediated and humoral antibody response and has antimicrobial and anti-inflammatory functions. Its deficiency may be related to cognitive impairment and dementia and increases the risk for acquiring several infectious diseases [24]. In patients with EDs vitamin D3 deficiency has been correlated with osteoporosis risk, while in patients with long-term EDs might be responsible for the lack of the inflammatory response and the depressive symptoms [25]. Interestingly, in our study a significant (p < 0.0001) correlation between lymphocytes count, body weight, BMI, and some vitamins (B1, B6, C and D3) was found. The correlation between lymphocytes number, vitamin C and vitamin D leads to assume that these patients have a higher susceptibility to infections. Moreover, in the literature it is reported that for many patients with malnutrition one of the key outcomes is amenorrhea [20]. In our study we found that the amenorrhea affected a high percentage of females (64%) and that these patients, in addition to low body weight, had lymphocytopenia. On this basis we can assume that in these patients the amenorrhea may be an adaptive, but completely reversible condition to malnutrition [20].

In adolescent, an excellent indicator of nutritional status is serum concentration of thyroid hormones, being they influenced by both the degree of leanness and the current weight trend. In our study, we found that 7.9% of ED patients had high levels of thyrotrophic hormone (TSH) and that 21.3% of patients had low levels of thyroxine (FT4). Increased levels of TSH and low levels of FT4 are typical of hypothyroidism, most often caused by autoimmune thyroid disease such as Hashimoto’s thyroiditis (HT). According to the literature data we found that TSH correlated with some vitamins such as vitamin B1 (rho = 0.025), vitamin B6 (rho = 0.21) and vitamin D3 (rho = 221 0.056) and total cholesterol content (rho = 0.127) [26–29]. Moreover, we found that a small percentage of patients (11.8%) had total cholesterol values higher than the reference values (median values 244.6 mg/dL). This could have important clinical implications and become a risk factor for cardiovascular disease. In addition, in many patients (56%) high serum ferritin levels were found. This is probably due to an increase in muscle catabolism that occurs during the loss of the menstrual cycle [30]. All these results show that there are frequent comorbidities in eating disorders, mainly related to malnutrition, which need to be analyzed.

## Conclusions

The COVID-19 pandemic has negatively affected teens’ eating behavior. The data obtained in this retrospective study show that adolescents who experienced eating disorders during the pandemic, often present comorbidities, and alterations of blood parameters such as leukocytopenia, neutropenia, hypovitaminosis and hormonal problems that could danger their future.

These comorbidities should not be overlooked, but they should be treated in association with psychological treatments.

## Electronic supplementary material

Below is the link to the electronic supplementary material.

Supplementary Material 1

## Data Availability

The datasets used and/or analyzed during the current study are available from the corresponding author on reasonable request.

## Abbreviations

AN: Anorexia nervosa
ARFID: Avoidant/restrictive food intake disorder
BED: Binge-eating disorder
BMI: Body mass index
BN: Bulimia nervosa
EDs: Eating disorders
FSH: Follicle- stimulating hormone
FT4: Thyroxine
HT: Hashimoto’s thyroiditis
SLD: Specific learning disorder
TSH: Thyrotrophic hormone
