# Supplementary material for: The impact of the COVID-19 pandemic on eating disorders risk and symptoms: a retrospective study
Source: Ital J Pediatr. 2023 Apr 26;49:50. doi: 10.1186/s13052-023-01443-6 (PMC10132438; doi:10.1186/s13052-023-01443-6)
Supplement: Supplementary file 1 — Supplementary Material 1 [file 13052_2023_1443_MOESM1_ESM.docx]

Rome, March 7, 2023

**Dear Editor,**

**Enclosed please find the revised manuscript ITJP-D-22-00434R3** **entitled “The impact of the COVID-19 pandemic on eating disorders risk and symptoms: a retrospective study” by** Isabella Tarissi De Jacobis, Teresa Capriati, Italo Pretelli, Annalisa Grandin, Cristina Mascolo, Rosa Vona, Lucrezia Gambardella, Camilla Cittadini, Alberto Villani, Maria Rosaria Marchili **and** myself, submitted for publication as research manuscript in *Italian Journal of Pediatrics.*

This manuscript fits the purpose of the journal.

It describes the clinical conditions of a group of Italian adolescents who, during the COVID-19 pandemic, were affected by eating disorders.

I confirm that neither the manuscript nor any parts of its content are currently under consideration or published in another journal.

All authors have approved the manuscript and agree with its submission to *Italian Journal of Pediatrics.*

I also declare no conflict of interest.

Elisabetta Straface

Biomarkers Unit, Center for Gender-Specific Medicine

Istituto Superiore di Sanità

Viale Regina Elena 299 - 00161 Rome, Italy

Phone +39 06 49902443; Fax +39 06 49903691

Email:elisabetta.straface@iss.it

**Point by point**

**Reviewer #3:**

**Dear authors,
your paper is of high importance. However, there are moments which need to be clarified.**

**1. Leukocytopenia, neutropenia, hypovitaminosis and hormonal problems - these changes observed in patients. Are they connected to the certain comorbidities or not? It's recommended to do a regression analysis to see the connection between parameters.**

Authors: Thanks for this remark. As suggest a regression analysis was performed to connect hematological changes detected in ED patients with comorbidity such as bradycardia, amenorrhea, anxiety, and depression, but no correlations were found.

**2. Number of patients is too low.**

Authors: His consideration is exact. In this study 230 patients with EDs were enrolled, but only patients (127 patients) who had a follow-up after 1 month, 3 months and, in some cases, 12 months of antipsychotic therapy were evaluated.

**3. It is now really clear out of your conclusions what is new does your study bring to the field. Please clarify your conclusions.**

Authors: The conclusions have been clarified.

**4. In "Discussion" section you need in more clear and structured way to compare own results and already known data.**

Authors: The “Discussion” section was revised.
